# Nasal Cytokine Profiles of Patients Hospitalised with Respiratory Wheeze Associated with Rhinovirus C

**DOI:** 10.3390/v11111038

**Published:** 2019-11-07

**Authors:** Chisha T. Sikazwe, Ingrid A. Laing, Allison Imrie, David W. Smith

**Affiliations:** 1 PathWest Laboratory Medicine WA, Perth 6009, Australia; 2 Faculty of Health and Medical Sciences, University of Western Australia, Perth 6009, Australia; allison.imrie@uwa.edu.au; 3 Telethon Kids Institute, Perth 6009, Australia; ingrid.laing@telethonkids.org.au

**Keywords:** rhinoviruses, infection, immunity, wheeze, viral load

## Abstract

Background: Rhinovirus C is an important pathogen of asthmatic and non-asthmatic children hospitalised with episodic wheeze. Previous studies on other respiratory viruses have shown that several host cytokines correlate with duration of hospitalisation, but this has yet to be investigated in children with RV-C infection. We determined the nasal cytokine profiles of these children and investigated their relationship with RV-C load and clinical outcome. Flocked nasal swabs were collected from children aged 24–72 months presenting to the Emergency Department at Princess Margaret Hospital with a clinical diagnosis of acute wheeze and an acute upper respiratory tract viral infection. RV-C load was determined by quantitative RT-PCR and cytokine profiles were characterised by a commercial human cytokine 34-plex panel. RV-C was the most commonly detected virus in pre-school-aged children hospitalised with an episodic wheeze. RV-C load did not significantly differ between asthmatic and non-asthmatic patients. Both groups showed a Th2-based cytokine profile. However, Th17 response cytokines IL-17 and IL-1β were only elevated in RV-C-infected children with pre-existing asthma. Neither RV-C load nor any specific cytokines were associated illness severity in this study. Medically attended RV-C-induced wheeze is characterised by a Th2 inflammatory pattern, independent of viral load. Any therapeutic interventions should be aimed at modulating the host response following infection.

## 1. Introduction

Rhinoviruses (RVs) are the most frequently detected viruses in pre-school-aged children (3–5 years) hospitalised with respiratory wheeze [1,2]. Rhinovirus C (RV-C) is disproportionately the most commonly detected of the three RV species and is of particular importance because it is strongly associated with asthma-related hospitalisations [1,3,4]. RV-associated early wheezing episodes are an independent risk factor for recurrent wheezing and asthma inception [5,6]. Factors that contribute to RV-associated wheezing illnesses have not been completely elucidated but appear to have little or no association with viral load [7]. Evidence is accumulating that the host immune response may be the most important factor in predicting clinical outcome following infection [8,9]. However, little data exist on the nasal cytokine profile of hospitalised children following RV-C infection. The cytokine profiles of other respiratory viruses have been associated with duration of hospitalisation [10], but to our knowledge this is yet to be described for RV-C. There are some data to suggest that asthmatic patients infected with RV respond differently than non-asthmatic patients. Human challenge studies using RV-16 (a genotype within the RV-A species) reported differences in cytokine profiles between asthmatic and non-asthmatic patients [11], and RV-B infection is associated with lower levels of cytokine production and less severe respiratory symptoms compared to the other RV species [12]. Little data exist on the differences in cytokine profiles of asthmatic and non-asthmatic patients presenting to hospital with RV-C infection. This study was undertaken to characterise the nasal cytokine profile and viral load in these children with the aim of determining the role of viral replication and host responses in the pathogenesis of RV-C-induced wheeze.

## 2. Materials and Methods 

### 2.1. Study Cohort and Material Collection

Patients aged 24–72 months with a clinical diagnosis of wheeze accompanied by signs and symptoms of an upper respiratory tract viral infection were recruited from Princess Margaret Hospital for Children, Perth, Western Australia, in the Emergency Department (ED) and inpatient wards between June 2012 and June 2015. Exclusion criteria included oxygen saturation (SpO2) of 91% or less in room air at presentation, features of critical wheeze (silent chest on auscultation or exhaustion with or without cyanosis), oral corticosteroid therapy within the last 14 days, previous enrolment in the current study, clinical evidence of shock or bacterial sepsis, premature birth (<34 weeks gestation), known underlying cardiac or other respiratory disease, continuing immunosuppressant therapy or known to have immunodeficiency, upper respiratory tract structural abnormality, active varicella infection, known allergy to prednisolone, high clinical suspicion of an alternative diagnosis for wheeze (i.e., inhaled foreign body), and previous intensive care unit admission with wheeze or asthma. Children who were confirmed as meeting the criteria were swabbed in each nostril using a nasopharyngeal (NP) swab (FLOQSwabs™, Copan Diagnostics, Murrieta CA, USA), which was then placed in 5 mL of virus transport media, transported at 4–8 °C, and stored at −80 °C prior to testing. Asthma classification was based on the clinical judgment of the ED physician abiding by the Princess Margaret Hospital for Children clinical guidelines, which are based on the Global Initiative for Asthma (GINA) Global Strategy for Management and Prevention (GINA).

A subset of patients that were diagnosed with RV-C-associated wheeze and were representative of the study populations for sex ratio and asthma profile were chosen for cytokine studies. Healthy controls for the cytokine studies comprised 10 samples from community children attending local immunisation clinics, who had no evidence of an acute respiratory tract infection or of a chronic respiratory illness, and who were matched to cases on age and sex. The swabs from control children were collected, stored, and tested in the same manner as NP swabs from cases.

### 2.2. Nucleic Acid Extraction and PCR and Sequencing

The NP swabs in VTM were thawed and vortex-mixed. Nucleic acid was extracted from 200 µL of the VTM using the semi-automated MagMAX magnetic bead-based purification system (ThermoFisher, Sydney NSW, Australia). Nucleic acid was detected using a multiplex double real-time RT-PCR for influenza A, B, and C viruses (FLUA, FLUB, FLUC), parainfluenza virus 1–4 (PIV), RSV-A and -B, coronaviruses (hCoV-HKU1, -NL63, -OC-43, -229E), human metapneumovirus (hMPV), human bocavirus (hBoV), and human adenoviruses (hAdV) [13]. Every multiplex PCR run performed included positive controls for the nucleic acid extraction, for the PCR reaction, and for inhibitor detection, and included negative controls after every 5th sample to detect contamination. RV screening and genotyping was completed as previously described [1]. Briefly, primers targeting a 260 bp region in the 5′ untranslated regions (UTR) of RV were used to perform a semi-nested PCR. Second-round PCR product was visualised by gel electrophoresis. PCR product was treated with ExoSAP-IT (Thermo Fisher Scientific, Australia) prior to sequencing using the BigDye Terminator v.3.1 cycle sequencing kit (Thermo Fisher Scientific, Australia). RV species C assignment was confirmed by sequencing of RV positive samples at the 420 bp VP4/2 coding region as previously described [14]. Briefly, cycle sequencing was performed using the BigDye Terminator v.3.1 cycle sequencing kit (Thermo Fisher Scientific, Australia) according to manufacturer’s protocol. Following cycle sequencing, unincorporated dye terminators and salts were purified using the AxyPrep Mag PCR Clean-Up kit (Axygen, Corning NY, USA) according to the manufacturer’s protocol. Capillary sequencing was performed on the Applied Biosystems 3730xl DNA Analyzer (Thermo Fisher Scientific, Australia). Geneious (Geneious, Auckland, New Zealand) was used for sequence data analysis and standard nucleotide BLAST searches against virus families, bacteria and cell sequences from the Genbank. Viral load determination was completed on the Rotor Gene 6000 cycler (QIAGEN, Melbourne VIC, Australia) [15]. All experiments, including positive controls and negative controls, were completed in triplicate. Detection of the human gene for glyceraldehyde 3-phosphate dehydrogenase (GAPDH) mRNA [16] was utilised to ensure adequate specimen collection, RNA extraction, and detection of PCR inhibitors. 

### 2.3. Cytokine Measurement 

Measurements of interferon gamma (IFN-γ), IL-12p70, IL-13, IL-1β, IL-2, IL-4, IL-5, IL-6, TNF-α, GM-CSF, IL-18, IL-10, IL-17A, IL-21, IL-22, IL-23, IL-27, IL-9, IFN-α, IL-31, IL-15, IL-1α, IL-1RA, IL-7, TNF-β, eotaxin, GRO-a, IL-8, IP-10, MCP-1, MIP-1b, SDF-1a, RANTES, and IL-29 in a human cytokine 34-plex panel was completed commercially (Jomar Life Research, Melbourne VIC, Australia). These were chosen to include cytokine responses characteristic of the Th1, Th2, and Th17 pathways [17], and to include the innate immune response. 

### 2.4. Statistical Analysis

Data were not normally distributed and were therefore assessed non-parametrically. The Mann–Whitney U test was applied to compare two groups, and the Kruksall–Wallis test was applied when comparing three or more groups. Non-parametric Spearman’s rank coefficient was used for correlations. Results in text and tables are presented as medians with the interquartile range. In all statistical analysis, a *p*-value <0.05 was considered statistically significant. All statistical analyses were carried out using SPSS statistical software version 16.0 (IBM Inc Chicago, USA).

### 2.5. Ethics

As all patients receiving medical treatment for their symptoms were children below the age of consent, informed consent for the testing of the swab and downstream sample processing was given by the child’s parent or legal guardian. All methods and experimental protocols were approved by the Princess Margaret Hospital for Children Human Ethics Committee and the Research Governance Office (project numbers 1970/EP and 2014117EP).

## 3. Results

### 3.1. Virus Detections

**Figure 1:**
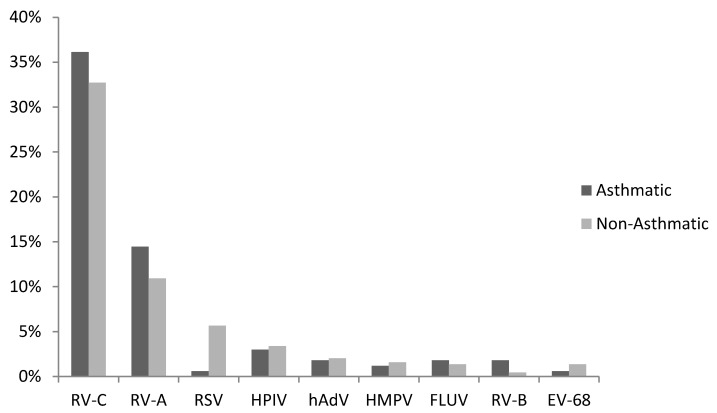
Respiratory virus detection rates in children presenting to the ED with a respiratory wheezing illness. RV-C was the predominant virus pathogen detected. Abbreviations: rhinovirus (RV), respiratory syncytial virus (RSV), human metapneumovirus (hMPV), adenovirus (ADV), human parainfluenza virus (HPIV), influenza viruses (IFV), human coronavirus (hCoV), and HBoV (human bocavirus).

The study enrolled 605 children aged between 24 and 72 months presenting to the ED with respiratory wheeze. Of these, 162 (27%) were classified as asthmatic and the remaining 443 (73%) were classified as non-asthmatics at the time of the study. Of the 605 cases screened for a respiratory virus, 65% (*n* = 393) returned a positive test result. Rhinovirus (RV) (69%), respiratory syncytial virus (RSV) (7%), and human parainfluenza virus (hPIV) (5%) were the most frequent (Figure 1). Speciation of RV positive cases clearly showed that RV-C (207/271, 76%) was the most commonly detected rhinovirus species followed by RV-A (64/271, 24%). Interestingly, RV-C was the sole pathogen detected in 92% (*n* = 191) of the RV-C positive samples.

### 3.2. RV-C and Asthma Diagnosis

When the analysis was stratified for physician-diagnosed asthma and as shown in Figure 1, RV-C and RV-A remained the predominant viruses detected and were both as common in the asthmatic as in non-asthmatic children. RSV was significantly (*p* = 0.02) less common in the asthmatic group compared to the non-asthma group. 

The analysis also compared viral load in asthmatic and non-asthmatic children infected with RV-C alone. As can be seen in Table 1, RV-C viral load was the same in the two groups, and there was no statistically significant difference in the length of hospital stay (Table 1). 

### 3.3. Cytokine Response Following RV-C Infections

A subset (*n* = 33) of the main study population where RV-C was the only detected pathogen and representative of the study’s sex ratio and the asthma profile were chosen to characterise the cytokine response in RV-C-associated wheeze. For these samples, the screening of RV-C determined by 5‘-UTR sequencing was confirmed by sequencing of the VP4/2 coding region. Of the 33 samples selected for the cytokine studies, 18 were RV-C positive patients with asthma and 15 were RV-C positive patients not diagnosed with asthma. None of the 33 patients received steroids at presentation (and, as per inclusion criteria, had not received oral corticosteroid therapy within the last 14 days) and were detected with an RV-C-only infection. Ten nasal samples from otherwise healthy community controls matched for age and sex were included in this part of the study; however, only five were included in the analysis as the other five tested positive for RV (*n* = 4) or RSV (*n* = 1).

**Figure 2:**
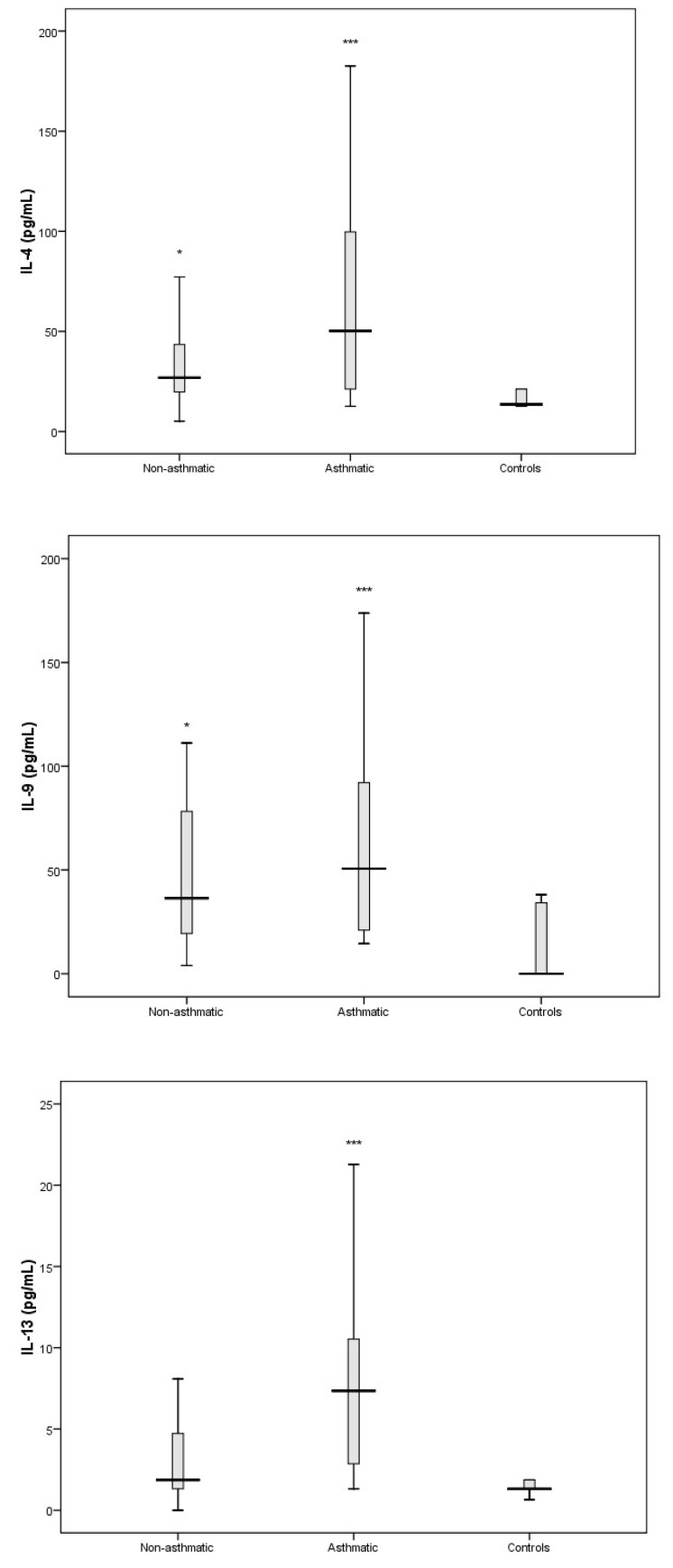
Levels of Th2 cytokines IL-4, IL-9, and IL-13 in nasal secretions of RV-C-infected asthmatics, RV-C-infected non-asthmatics, and healthy controls. * *p* < 0.05; *** *p* < 0.001.

The production of chemokines and cytokines representative of Th1, Th2, and Th17 responses were examined in children with asthma and compared to that of healthy controls. The Th2-associated cytokines IL-4, IL-9, and IL-13 (Figure 2) were all significantly elevated in RV-C-infected children with a diagnosis of asthma; however, the levels of Th2-associated cytokine IL-5 showed no significant difference compared to that of the healthy control group (Table S1).

**Figure 3:**
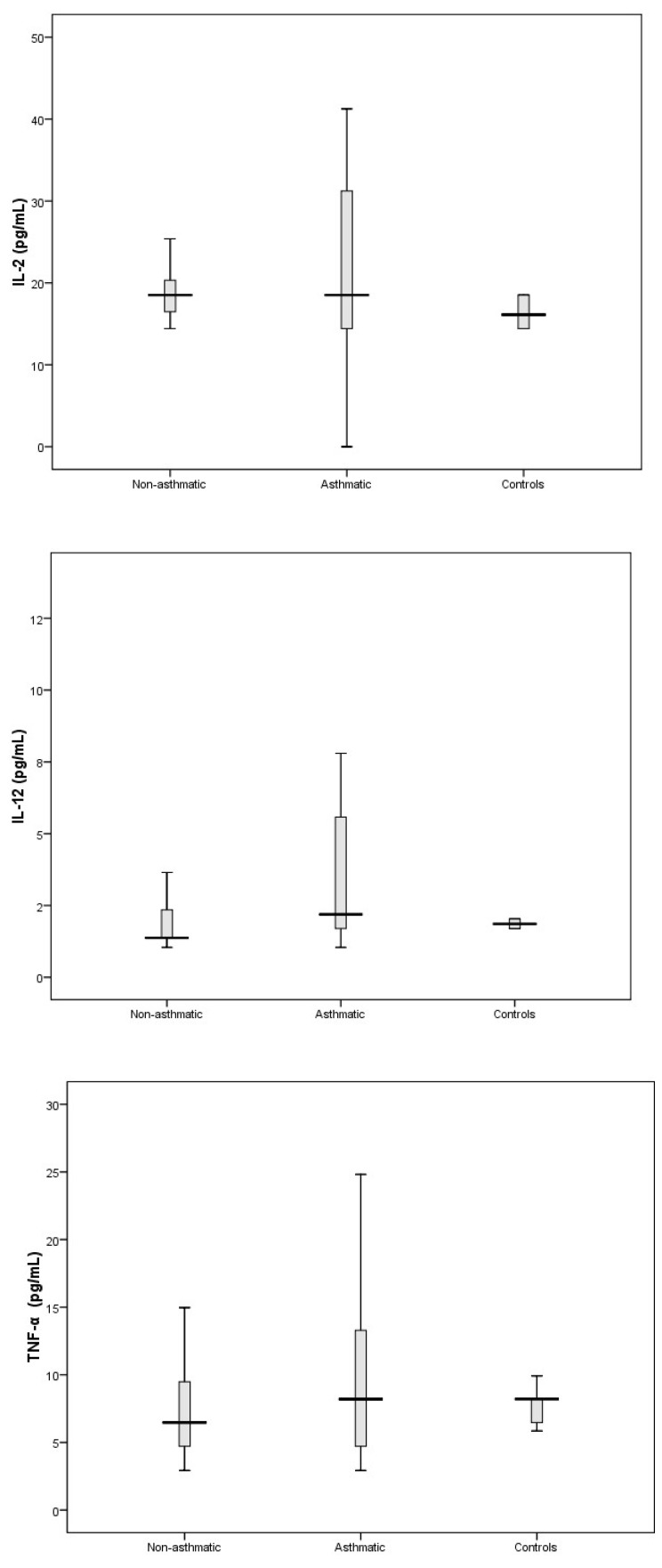
Levels of IL-2, IL-12, and TNF-α in nasal secretions of non-respiratory disease controls, and in RV-C-infected patients with asthma and without asthma. Levels of TNF-α, IL-2, and IL-12 did not significantly differ when each group (asthmatics and non-asthmatics patients) was individually compared to controls.

Levels of Th1-associated cytokines IL-2, IL-12, and TNF-α (Figure 3) showed no significant difference compared to controls.

**Figure 4:**
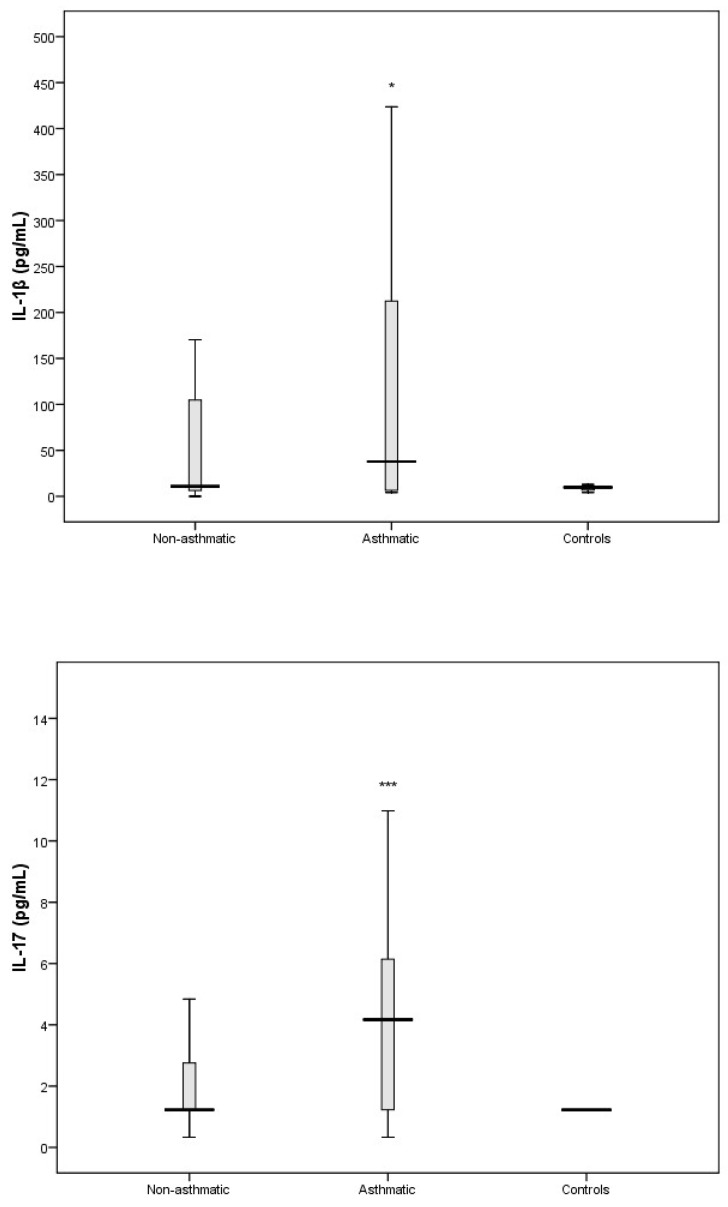
Levels of IL-1β and IL-17 in nasal secretions of non-respiratory disease controls, and RV-C-infected patients with asthma and without asthma. IL-1β and IL-17 were only elevated in the asthma patient group compared to controls. * *p* < 0.05, *** *p* < 0.001.

**Figure 5:**
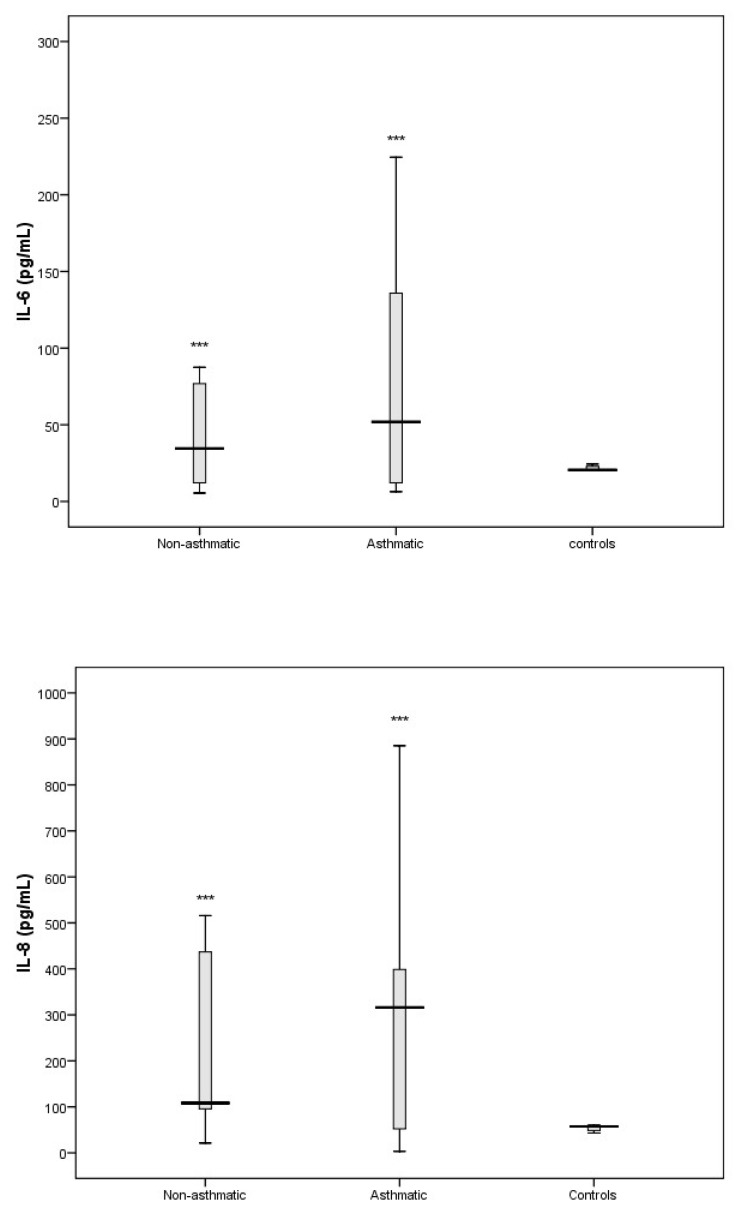
Levels of IL-6 and IL-8 in nasal secretions of non-respiratory disease controls (*n* = 5), RV-C-infected patients with asthma (*n* = 18 and without asthma. IL-6 and IL-8 were both significantly elevated both patient groups compared to controls. *** *p* < 0.001.

**Figure 6:**
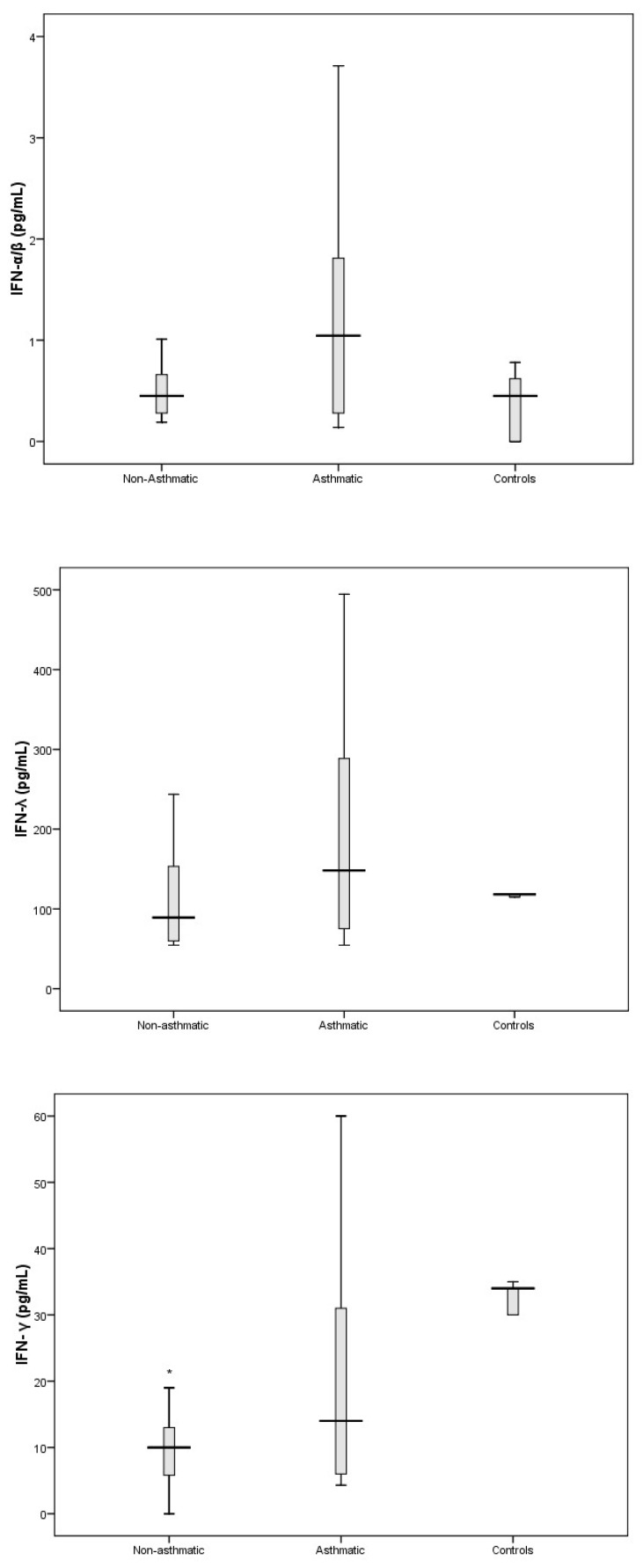
Levels of interferon (IFN)- α, IFN-λ, and IFN-γ in nasal secretions of controls, and hospitalised RV-C-infected patients with asthma and without asthma. IFN-γ was attenuated in the non-asthmatic group to controls. In RV-C-infected children with preexisting asthma, there was a trend towards reduced IFN-γ levels (*p* = 0.08). The levels of IFN-λ, and IFN-α were not significantly different in either patient group compared to controls. * *p* < 0.05.

The cytokines that comprise part of the Th17 response (IL-1β and IL-17A) and are important in promoting the secretion of pro-inflammatory cytokines were significantly elevated in RV-C-infected children with asthma (Figure 4) compared to controls. We also found that the pro-inflammatory cytokines IL-6 and IL-8 were elevated in RV-C-infected children with asthma (Figure 5) when compared to controls. In contrast, the levels of interferons I, II, and III, represented by IFN-α/β, IFN-γ, and IFN-λ, respectively, showed no significant difference compared to the control group (Figure 6).

Overall, RV-C-infected children with pre-existing asthma displayed a cytokine profile characterised by elevated levels of Th2, Th17, and pro-inflammatory responses. However, cytokine levels of Th1 responses and interferon responses displayed no significant changes compared to healthy controls. 

In this study we were also interested in characterising the nasal cytokine profile of RV-C-infected children without pre-existing asthma. The Th1-associated cytokines IL-2, IL-12, and TNF-α showed no significant differences when compared to controls (Figure 3). Furthermore, IFN-γ (an important contributor to the clearance of viral infections) was significantly attenuated in RV-C-infected children without a diagnosis of asthma, whereas IFN-α/β and IFN-λ showed no significant differences when compared to controls (Figure 6). The Th2-associated cytokines IL-4 and IL-9 (Figure 2) were significantly elevated in RV-C-infected children, but IL-5 (Table S1) and IL-13 (Figure 2) showed no significant difference when compared to controls. The levels of Th17-associated cytokines IL-1β and IL-17A (Figure 4) showed no significant difference compared to healthy controls, but the levels of pro-inflammatory cytokines IL-6 and IL-8 were significantly elevated compared to controls (Figure 5).

Taken together, our data demonstrate that the cytokine profile in children without pre-existing asthma is characterised by increased levels of Th2 and pro-inflammatory cytokines, a reduction in IFN-γ, and unchanged levels in IFN α/β, Th1, and Th17 cytokines.

### 3.4. Relationships Between Cytokines, RV-C Load, and Clinical Outcomes

A univariate analysis was conducted to determine the influence of viral load on the cytokine responses. This was confined to inflammatory mediators that had shown a significant difference from the control group separately for asthma and non-asthma patient groups (Tables S2 and S3). These showed weak, non-significant relationships between the selected cytokines and RV-C load for both groups. Moreover, none of the cytokines analysed showed any association with the likelihood of hospitalisation (Table S4).

## 4. Discussion

Rhinoviruses have been shown to be commonly found in children presenting with wheezing illness, and there is increasing evidence of their importance as triggers of asthma exacerbations. In particular, RV-C has been implicated as a cause of wheezing illness in children with and without pre-existing asthma in Western Australia and elsewhere [3]. In this study, we looked at the viral pathogens present in children presenting at a paediatric emergency department with wheezing illness, and then looked at the role of pre-existing asthma, viral load, and the host inflammatory response in causing illness.

Rhinoviruses, especially RV-C, were the overwhelming viruses detected in these children, supporting their role as the major cause of respiratory wheezing illness in pre-school-aged children presenting to hospital. The proportion of RV in this group of children is similar to the range reported in the published literature internationally [18,19,20], and in a previous study in Western Australia [3]. The frequency of detection of the various respiratory viruses, including RV-C was the same in the children with and without pre-existing asthma, and the length of hospitalisation did not differ significantly between asthmatic patients and non-asthmatic patients. However, it was unusual for the children to spend more than a few hours at the hospital, and the median length of stay was only 10 h for the asthmatic group and 7 h for the non-asthmatic group, so it was difficult to demonstrate statistically significant differences in a study of this size. Moreover, as hospitalisation was very infrequent, we could not look at other inpatient outcome measures, and we did not have outpatient follow-up data. However our data do suggest that RV-C induces a relatively short clinical illness in asthmatic and non-asthmatic children with few severe complications. This is consistent with a recent multicentre study, which reported that children with a rhinovirus-only infection had shorter acute clinical course compared to children with RSV-only infection [21].

As RV-C is an important cause of wheeze in preschool-aged children, we looked at the inflammatory markers that may identify the pathogenic process and at how this may be influenced by pre-existing asthma in the child. Our results indicate that both asthmatic and non-asthmatic RV-C-infected children displayed an increase in Th2- and pro-inflammation-associated cytokines. IL-4 and IL- 9 were Th2 cytokines that were elevated in both groups of RV-C-infected children. However, the levels of these cytokines were higher in children with pre-existing asthma than in non-asthmatic children. Therefore, these Th2 cytokines can be considered as signature responses to RV-C infection in children presenting to hospital with wheeze. Both IL-4 and IL-9 are known to shape the immune response in allergic asthma. Previous studies have demonstrated that Th2 cytokine IL-4 mediates recruitment of inflammatory cells to the lung, IgE isotype class switching, upregulation of high affinity IgE receptors on mast cells and basophils, and IgE-dependent mast cell activation, which together result in the development of immediate allergic reactions and mucus hypersecretion [22,23]. IL-9 can elicit detrimental effects on epithelial integrity and ciliated cell differentiation leading to mucus hypersecretion as well as impaired mucus clearance [24]. It is also likely that the intrinsic immunologic predisposition of children with asthma amplifies an existing Th2 pulmonary environment [25], which may explain why the levels of Th2 cytokines were higher and broader (Figure 2) in RV-C-infected children with pre-existing asthma. Of note, elevated IL-13 was only seen in this group.

It was surprising that the Th2 cytokine response was dominant in children without pre-existing asthma. In the pulmonary environment of patients without known underlying chronic inflammation, we had expected a dominant early Th1 cytokine response with elevation of antiviral proteins capable of suppressing viral replication. However, the levels of Th1 cytokines were either significantly attenuated (IFN-γ and IL-18) or showed no differences (IL-12, IFN-α, and IFN-λ) when compared to controls. Further, we did not observe any difference in viral load when patients with pre-existing asthma were compared to those without. It is quite possible, therefore, that medically attended RV-C infection in children promotes the induction of a Th2 cytokine response that in turn dampens the antiviral cytokine response resulting in delayed viral clearance and prolonged airway inflammation [8,26].

Interestingly, Th17 response cytokines IL-17 and IL-1β were only elevated in RV-C-infected children with pre-existing asthma. Previous studies demonstrated high Th17 responses in the airways epithelium of patients with severe asthma [27]. IL-17 is known to facilitate neutrophil activation and proliferation in neutrophilic asthma, in part by enhancing the production of the pro-inflammatory cytokines IL-6 and IL-8 [28,29]. Additionally, previous studies have shown that IL-17 enhances the production of Th2 cytokine IL-13 and results in airway hyper-responsiveness, airway inflammation, and mucus hypersecretion [30,31]. IL-1β is released following RV infection and contributes to pathogenesis of respiratory disease by recruitment of inflammatory cells and enhancing production of IL-8 [32]. Further, experimental human models of asthma exacerbations demonstrate that an early rise in IL-1β in respiratory secretions is temporally associated with clinical symptoms [32,33]. In the present study, we found evidence that pro-inflammatory cytokine responses (IL-6 and IL-8) were broader and stronger in the children with pre-existing asthma. We also found that only RV-C-infected children with pre-existing asthma had elevated levels of Th2 cytokine IL-13. Taken together, our data suggest that Th17 cytokines are central to the immune response following RV-C infection in children with asthma and may be responsible for some features of airway inflammation observed in this patient group.

This study has potential limitations. Firstly, only one time point was used for the measurement of cytokines and viral load, and although early evaluations are useful for the acute phase response, they do not permit further insights such as those provided by sequential measurements. Even though it is tempting to speculate that there is a direct association between cytokine concentrations and illness severity, the full burden of disease cannot be solely attributed to this phenomenon because cytokines may behave as markers of tissue damage, without necessarily contributing to pathology directly. We cannot exclude the possibility that children classified as non-asthmatic in this study may in fact be asthmatic and not yet been diagnosed; therefore, the observed dominance of Th2-type responses following RV-C infection in “non-asthmatics” may in fact be a marker of predisposition to asthma. Diagnosing asthma in children below the age of six years, as in this study, can be difficult because episodic respiratory symptoms such as wheezing are very common in children in that age group. This study cannot conclusively state that cytokine responses specific to RV-C since we did not look at cytokine responses in patients with either RV-A or RV-B infection. Lastly, for the epidemiological component of the study we used 5′UTR sequence for species assignation because it provided the greatest sensitivity and minimised the risk of sampling bias in our study. Our previous experience [34,35] as well as data from other studies [36,37] have shown a high correlation between the two methods. However, for the critical pathogenicity studies, we did confirm the species by VP 4/2 sequencing.

In summary, RV-C is the predominant pathogen-associated with respiratory wheeze in hospitalised preschool-aged children in our population, causing a short-lived clinical illness. RV-C load is neither a risk factor nor a reliable marker for hospitalisation following infection. RV-C infection, of itself, is more important than the level of viral replication in determining the clinical illness, as factors other than viral load drive clinical course. RV-C-associated respiratory wheeze in hospitalised children is characterised by a dominant Th2-type inflammatory response and suggests a synergistic relationship between RV-C and host factors in the genesis of wheeze. Furthermore, RV-C promotes a more intense cytokine response in children with asthma compared to children without asthma. The induction of Th17 cytokines that mediate recruitment and activation of neutrophils may be an important underlying pathogenic mechanism associated with RV-C disease in children with pre-existing asthma. Current and future therapeutic interventions should be aimed at modulating the host response following infection.

## Figures and Tables

**Table 1:** Summary of clinical and demographic data of hospitalised children with RV-C-only respiratory wheeze (*n* = 191).

	Asthmatic	Non-Asthmatic	*p*-Value
Age (mean ± SD)	2.9 ± 1.2	2.8 ± 1	n.s. ^#^
Sex (male %)	78	73	n.s
History of Eczema (%)	45	20	<0.01
History of Hay Fever	11	0	<0.0001
Median RV-C Log10 (copies/mL, IQR)	5.8 (3.1–6.4)	5.8 (3.8–6.8)	n.s. ^#^
Length of Stay (median hours, IQR)	7 (1–16)	10 (2–16)	n.s. ^#^

# Comparison of continuous variables (independent samples *t*-test/Mann–Whitney U test); comparisons of proportions (Fisher’s exact test); n.s.: non-significant; SD: standard deviation; IQR: interquartile range.

## Supplementary Materials

The following are available online at https://www.mdpi.com/1999-4915/11/11/1038/s1, Table S1: Nasal cytokine levels of healthy non-respiratory disease controls, RV-C infected patients with asthma and without asthma; Table S2: The relationship between RV-C load and inflammatory mediator production in the nasal secretions of children with asthma; Table S3: The relationship between RV-C load and inflammatory mediator production in the nasal secretions of children without asthma; Table S4: Association between cytokine production and hospitalisation of children hospitalised following RV-C infection using the Mann-Whitney U test.
