# Supplementary material for: Nasal Cytokine Profiles of Patients Hospitalised with Respiratory Wheeze Associated with Rhinovirus C
Source: Viruses. 2019 Nov 7;11(11):1038. doi: 10.3390/v11111038 (PMC6893661; doi:10.3390/v11111038)
Supplement: Supplementary file 1 [file viruses-11-01038-s001.pdf]

Appendix A

Supplementary

Table S1. Nasal cytokine levels of healthy non-respiratory disease controls, RV-C infected patients with asthma and without asthma.

**Participants**	**IL-5**	**IL-9**	**IL-15**	**IL-18**	**IL-21**	**IL-22**	**IL-23**	**IL-27**	**IL-31**	**TNF-α**	**CXCL-12**	**CXCL-1**	**MIP-1β**	**RANTES**	**GM-CSF**	**Eotaxin**
Ctrls (n=5)	17	0	0	66	89	48	32	0	59	8	390	426	19	8	5	10
IQR	13-16	0-36	0-2	62-78	88-97	45-53	13-34	0-91	56-67	6-9	358-458	344-676	17-26	7-9	5-7	9-17
Asth (n=18)	12	51	10	38	114	157	38	103	77	8	331	193	45	9	7	8
IQR	7-23	20-95	6-19	12-76	32-191	45-389	16-99	25-241	31-147	5-15	124-453	93-315	17-67	3-21	2-15	5-14
Non-Asth (n=15)	14	36	7	22	50	91	16	47	38	5	142	103	25	4	5	6
IQR	10-16	18-87	4-10	13-26	29-81	35-174	15-35	11-107	23-55	5-11	79-228	62-240	10-87	3-5	2-8	5-9
p values																
Asth vs Ctrls	n.s	0.009	<0.001	n.s	n.s	0.048	n.s	0.005	n.s	n.s	n.s	0.002	n.s	n.s	n.s	n.s
Non-asth vs Ctrls	n.s	0.024	<0.001	0.004	0.025	n.s	n.s	0.047	0.025	n.s	0.005	0.001	n.s	0.007	n.s	0.004
Asth vs Non-asth	n.s	n.s	0.049	n.s	n.s	n.s	0.01	0.031	n.s	n.s	n.s	n.s	n.s	n.s	n.s	n.s

Abbreviations: IL-interleukin, TNF-α- Tumour necrosis factor alpha, CXCL- chemokine ligand, MIP- Macrophage Inflammatory protein, RANTES- Regulated on Activation, Normal T Cell Expressed and Secreted, GM-CSF-Granulocyte-macrophage colony-stimulating factor. Asth-asthmatics, Non.Asth-Non-asthmatics, Ctrls-controls, IQR- interquartile range.

Table S2. The relationship between RV-C load and inflammatory mediator production in the nasal secretions of children with asthma.

·	**IFN-α**	**IL-1β**	**IL-4**	**IL-6**	**IL-10**	**IL-17**	**IL-9**	**IL-15**	**CXCL-1**	**IL-8**	**IP-10**	**IL-22**	**IL-27**
Correlation (R^2)	0.05	-0.03	-0.10	-0.07	0.11	-0.06	-0.02	-0.08	-0.05	0.00	0.02	-0.18	-0.19
p- value	0.85	0.92	0.71	0.79	0.69	0.83	0.93	0.77	0.85	1.00	0.95	0.52	0.47

Table S3. The relationship between RV-C load and inflammatory mediator production in the nasal secretions of children without asthma.

	IFN-α	IL-4	IL-6	IL-8	IL-9	IL-15	IP-10	IL-18	IL-21	IL-27	IL-31	Eotaxin	CXCL-1	CXCL-12	RANTES
Correlation (R^2)	-0.16	0.14	-0.27	-0.01	-0.03	-0.29	-0.18	-0.31	-0.20	0.02	-0.19	-0.15	-0.06	-0.10	-0.42
p-value	0.59	0.64	0.35	0.97	0.92	0.32	0.53	0.28	0.50	0.95	0.51	0.62	0.83	0.74	0.13

Table S4. Association between cytokine production and hospitalisation of children hospitalised following RV-C infection using the Mann-Whitney U test.

**Cytokine**	**p-value (Asthmatics)**	**p-value (Non-Asthmatics)**
**IFN-γ**	0.26	0.947
**IL-4**	0.259	0.601
**IL-6**	0.212	1.00
**IL-8**	0.26	0.361
**IL-9**	0.109	0.744
**IP-10**	0.594	0.896
**IL-15**	0.191	0.512
**IL-27**	0.138	0.554
**CXCL-1**	0.515	0.794
**IL-13**	0.313	-
**IL-1B**	0.441	-
**IL-17**	0.233	-
**IL-22**	0.26	-
**IL-18**	-	0.794
**IL-21**	-	1.00
**Eotaxin**	-	0.946
**CXCL-12**	-	0.896
**RANTES**	-	0.647
**IL-31**	-	0.793

- denotes that analysis was not performed in that group.
